# Enhancement of Wound Healing and Angiogenesis Using Mouse Embryo Fibroblasts Loaded in Decellularized Skin Scaffold

**DOI:** 10.61186/ibj.3971

**Published:** 2024-04-06

**Authors:** Armaghan Gheytasvand, Hamed Bagheri, Shahram Pourbeyranvand, Mojdeh Salehnia

**Affiliations:** 1 Department of Biomaterials, Faculty of Interdisciplinary Science and Technology, Tarbiat Modares University, Tehran, Iran;; 2 Department of Anatomical Sciences, School of Medical Sciences, Tarbiat Modares University, Tehran, Iran

**Keywords:** Decellularized extracellular matrix, Neovascularization, Skin, Wound healing

## Abstract

**Background:**

Synthetic and natural polymer scaffolds can be used to design wound dressing for repairing the damaged skin tissue. This study investigated acute wound healing process using a decellularized skin scaffold and MEF.

**Methods:**

Mouse skin fragments were decellularized and evaluated by DNA content, toxicity, H&E staining, Raman confocal microscopy, Masson’s trichrome staining, SEM, and biodegradation assays. The fragments were recellularized by the MEFs, and cell attachment and penetration were studied. De- and decellularized scaffolds were used wound dressings in mouse acute wound models as two experimental groups. Using morphological and immunohistochemical assessments, wound healing was evaluated and compared among the experimental and control groups.

**Results:**

DNA content of the decellularized tissue significantly reduced compared to the native control group (7% vs. 100%; *p* < 0.05). ECM components, e.g. collagen types I, III, and IV, elastin, and glycosaminoglycan, were well preserved in the decellularized group. The porosity and fiber arrangement in the stroma had a structure similar to normal skin tissue. A significant reduction in healing time was observed in the group treated with a decellularized scaffold. A thicker epidermis layer was observed in the recovered tissue in both experimental groups compared to the control group. Immunostaining showed a positive reaction for CD31 as an endothelial marker in both experimental groups, confirming new vascularization in these groups.

**Conclusion:**

Using MEFs with decellularized skin as a wound dressing increases the rate of wound healing and also the formation of new capillaries. This system could be beneficial for clinical applications in the field of tissue engineering.

## INTRODUCTION

Wound healing is a complex and dynamic process that repairs damaged skin tissue. Currently, according to the types of wounds, various wound dressings have been developed and introduced to the market^[^^1^^,^^2^^]^. Wound dressings create a barrier to prevent the penetration of bacteria by covering the wound; however, they allows the penetration of gas molecules. They also provide some substrates for the growth and proliferation of cells; thus, the time of the wound-healing process would be decreased^[^^1^^]^. 

Different types of synthetic and natural polymer scaffolds derived from animal and plant tissues can be used to design wound dressings^[^^3^^-^^6^^]^. In this regard, natural scaffolds such as collagen, chitosan, and decellularized tissue have widely been used because of their biocompatibility, degradability, porosity, mechanical properties, and non-toxicity^[^^4^^]^. Decellularized scaffolds have been considered for the regeneration of damaged tissue by preventing chronic inflammation and immunological reactions owing to their macromolecular similarity to the body’s ECM^[^^1^^,^^4^^-^^7^^]^. Recellularization of the cell-free scaffold by seeding different cell types, especially stem cells, is regarded an ideal choice for reconstructing skin, bone, nerve, heart, lung, liver, and kidney^[^^6^^-^^8^^]^. 

In the literature, there are several studies on the use of DST as a wound dressing in different forms, such as tissue sheets, powders, gels, or fillers^[^^8^^-^^12^^]^. Compared to other scaffolds, decellularized tissue acts as a physiological reservoir for growth factors and cytokines that improve cell migration and proliferation during wound healing^[^^7^^-^^10^^]^. Moreover, this type of scaffold facilitates tissue repair by maintaining an ECM architecture similar to that of native tissue^[^^10^^]^. 

Numerous investigations have focused on the use of recellularized scaffolds by different cell types such as adult fibroblasts, keratinocytes, fat-derived stem cells, bone marrow stem cells, and umbilical cord perivascular cells^[^^6^^,^^13^^-^^18^^]^. MEFs are mesenchymal-derived cells that are capable of proliferating and differentiating into several cell types. Moreover, as the main component of connective tissue, these cells have different functional and morphological roles, especially in the repair of damaged tissue^[^^18^^-^^20^^]^. Thus, this study was designed to evaluate skin wound healing in a mouse model by transplantation of a decellularized scaffold with MEFs.

## MATERIALS AND METHODS

All materials and reagents were purchased from Sigma-Aldrich (London, UK), except for those noted.

**Collecting skin samples**

The adult male NMRI mice (n = 5) were killed by cervical dislocation, and their skin was removed, dissected into fragments and washed thoroughly in PBS. 

**Study design**

**Fig. 1:**
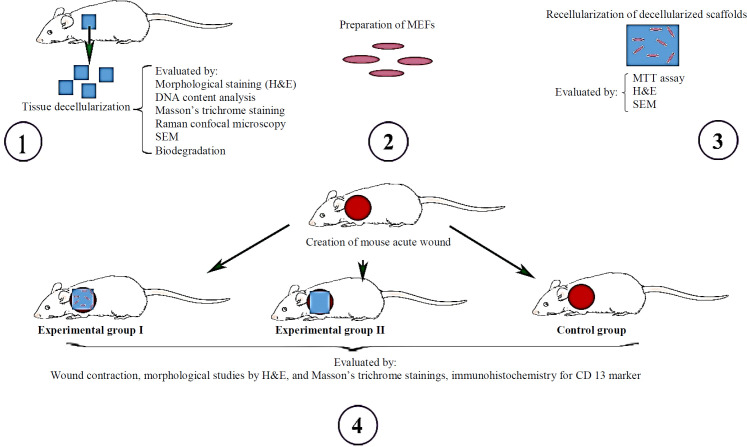
Study design

A summary of the study design is shown in Figure 1. First, the mouse skin was decellularized, and its quality was confirmed, and then cell seeding was performed using MEFs. Cell attachment and penetration within this scaffold were studied by H&E staining and SEM. Finally, after the establishment of the mouse model of an acute wound, the in vivo study was performed in two experimental groups I and II using the decellularized scaffold with or without MEFs as wound dressings, respectively. The wound healing was then evaluated and compared with the control group by morphological and immunohistochemical studies. 

**Decellularization of the skin tissue**

Before processing, the skin samples were cut into 1 × 2 cm^2 ^fragments. The tissues were immersed in EDTA on a shaker at 50 rpm at room temperature for 8 hours. After washing in PBS, the samples were treated with a solution containing 1% (w/v) Triton X-100 in a rotator shaker (50 rpm) at room temperature for 15 hours. Then they were washed in PBS and treated with 1% SDS in a shaker (50 rpm) at room temperature for 72 h. Finally, the samples were washed and sterilized by PBS containing penicillin-streptomycin and ethanol for 3 hours, respectively and stored at 4 °C^[^^21^^,^^22^^]^. Aseptic conditions were maintained throughout all steps of the protocols. 

**Morphological study of the decellularized tissue**

For histological evaluation, fresh (native) and decellularized tissue samples (n = 5, fragments in each group) were fixed in 10% formalin for 48 h, embedded in paraffin, and sectioned at 4-6 μm thickness. Two sets of tissue sections were prepared and stained using the H&E and Masson’s trichrome methods. 

**SEM analysis**

To evaluate the fine structure of mouse skin after decellularization, the cell-free and control samples (n = 3 in each group) were fixed in a solution of 2.5% glutaraldehyde in PBS and then dehydrated in increasing concentrations of ethanol. Finally, they were observed under a scanning electron microscope (VEGA/TESCAN-XMU, Czech Republic) after coating with gold particles^[^^17^^]^.

**DNA extraction**

The residual DNA content in fresh mouse skin and DST samples (n = 3 in each group) was determined using a commercial kit (TRIzol®; Invitrogen, UK) based on the manufacturer’s instructions. Briefly, the samples (n = 3 fragments in each group) were digested by TRIzol® reagent and then centrifuged. The supernatant was removed, chloroform was added, and the middle part of the centrifuged material was collected. In the next step, absolute ethanol and sodium citrate were sequentially added. Finally, the samples were treated with 75% ethanol and resuspended in NaOH. After centrifugation, DNA content was measured using UV spectrophotometry (Eppendorf, Germany) at an optical density of 260 nm. 

**In vitro biodegradability**

To do the biodegradability test, the decellularized scaffolds were dehydrated using ascending concentrations of ethanol, weighed and divided into two experimental and control groups (n = 6 in each group). In the control group, the decellularized tissue samples were placed in PBS, whereas in the experimental group, the decellularized specimens were placed in a solution consisting of 0.1% collagenase type I (Invitrogen, England). At intervals of 24 and 48 h, the samples in each group were dried and weighed again. Subsequently, biodegradability was calculated using the following equation: Degradation (%) = 100 × (W_0_-W_t_)/W_0_, in which W_0 _= initial weight and W_t _= weight of the tissue sample at time point t^[^^23^^]^.

**Raman confocal microscopy**

The decellularized and native samples (three samples in each group for three repetitions) were subjected to Raman confocal microscopy (Horiba, XploRA Plus, France). Excitation laser wavelength of 785 nm and a spectral range of 200-3500 cm^1^ were set up^[^^23^^,^^24^^]^. The amounts of glycosaminoglycan, collagen types I, III, and IV, and elastin in DST were compared with those in the control group.

**MEF preparation**

MEFs were obtained from the Cell Bank of the Anatomy Department at Tarbiat Modares University, Tehran, Iran. The cells were cultured in DMEM (Gibco, Thermo Fisher Science, USA) containing 10% FBS (Gibco, UK), 100 IU/ml penicillin, and 75 µg/ml streptomycin in 5% CO_2_ at 37 °C for 48 h. After confluency, the cells were trypsinized and used for the following assessments.

**Cell viability and cytotoxicity tests**

Cell proliferation and cytotoxicity for DST samples were determined by the MTT assay. The MEFs (3 × 10^5^) were placed into 96-well plates containing one fragment of the DST samples and cultured in 5% CO_2_ at 37 °C. After 24 and 72 h, 10 ml of MTT solution (5 mg/ml) was added into each well and incubated at 37 °C for 4 h. Next, the medium was removed and 600 μl dimethyl sulfoxide was added and kept for 15 min. The optical density values were read on a spectrophotometer at 570 nm.

**Recellularization of DST with MEFs**

MEFs were seeded onto the DST scaffolds at a density of 1 × 10^6^ cells/ml in DMEM supplemented with 10% (w/v) FBS and then incubated in 5% CO_2 _at 37 °C. After one week of cultivation, the samples were fixed, processed, stained and visualized by light microscopy. To evaluate cell attachment within the DST scaffolds, we fixed another series of samples and studied them by SEM as described earlier^[^^25^^]^.

**In vivo study**

Adult male mice with an average body weight of 25-30 g (n = 15) were anesthetized with an intraperitoneal injection of ketamine (80 mg/kg) and xylazine (5 mg/kg). Then a full-thickness cutaneous wound by skin punch with a diameter of 5 mm was created on the back of each mouse. Subsequently, the animals were randomly divided into experimental groups I and II, as well as control (n = 5 in each group). In the experimental group I, after the preparation of sterile scaffolds and recellularization with MEFs, they were used as wound dressings. In experimental group II, the surface of the created wound was covered by a fragment of the decellularized scaffold. In both experimental groups, a 10 × 10 mm-sized scaffold was applied to close the wound, and a cotton bandage was used as the second covering. In the control group, cotton bandage was used as the primary covering of the wound. The mice after the operation were kept under control conditions. 

**Wound contraction assessment**

Mice were monitored during healing by capturing images from their skin, and measuring the size of the wound surface. Wound contraction was evaluated as a reduction in the wound area and defined as the percentage closure of the wound surface on the healing day using this formula: T wound contraction (%) = A1- An A1 × 100, in which A1 = wound area on day 1 and An = wound area on day n^[^^11^^]^.

**Morphological studies of recovered tissue**

Morphological study of the wound healing was performed on the day of wound closure in each group. Samples were collected from the experimental groups I, II, and control on days 6, 8, and 21 after surgery, respectively. After cervical dislocation, tissue samples from the margin and the entire wound bed were harvested, fixed, passaged, embedded in paraffin wax and sectioned. The tissue sections were stained with H&E and Masson’s trichrome for histological examination. 

**Immunohistochemistry for the CD 13 marker in recovered tissue**

For CD 31 immunohistochemistry, another set of paraffin tissue sections was collected from both the experimental groups I and II and the control group. Briefly, the slides were deparaffinized in xylene for 20 min with two changes and hydrated by adding descending concentrations of ethanol and distilled water. After antigen retrieval, the endogenous peroxidase was inactivated by incubating the slides in a 3% H_2_O_2_ solution at room temperature for 10 min. Next, the samples were incubated in anti-mouse CD 31 primary antibody (1:200; Santa Cruz Biotechnology, USA) for 1.5 h, followed by incubation with secondary antibody conjugated with peroxidase (1:200; Santa Cruz Biotechnology) for 30 min. Finally, the slides were treated with diaminobenzidine and evaluated under a light microscope.

**In vivo tracking of labeled MEFs**

In another set of experiments, the presence of labeled MEFs in the recovered tissue after wound healing was evaluated in the experimental group II. Briefly, the MEF suspension (1 × 10^5^ cells/ml) was incubated with Hoechst 34580 solution (1 ng/ml) in the dark at 37 °C for 1 h. Finally, the cells were washed three times with PBS, and the labeled cells were used for recellularization as described earlier in section “Recellularization of DST with MEFs”. This decellularized scaffold was then used for wound dressing. After healing and preparing from the recovered samples, the tissue sections were observed under a fluorescence microscope at 370 and 535 wavelengths for Hoechst-labeled cells and PI-stained nuclei. 

**Statistical analysis**

Statistical analysis was performed using SPSS software (V24; SPSS Inc., Chicago, IL, USA). A one-way ANOVA test was used to analyze the data related to MTT and DNA content, and a student’s t-test was used for the analysis of biodegradation, Raman confocal microscopy, and wound contraction data. All results are presented as mean ± standard deviation, and p values <0.05 were considered statistically significant

## RESULTS

**Histological evaluation**

**Fig. 2:**
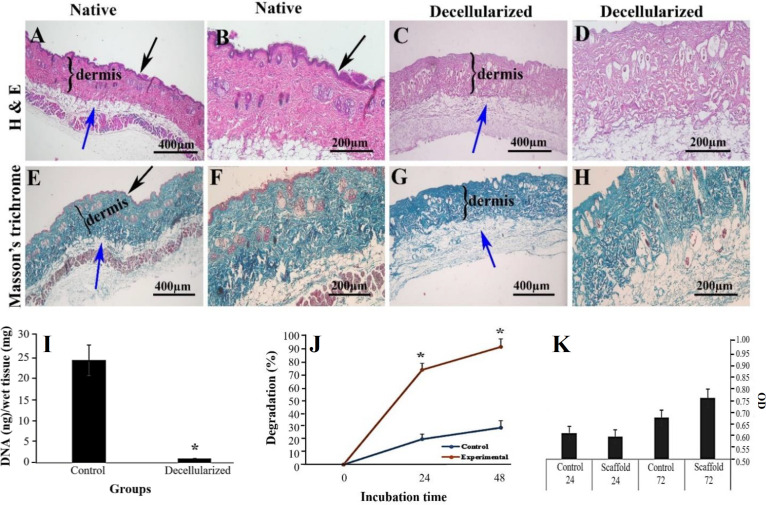
Morphology of mouse skin tissue sections after H & E staining. The normal (native) skin tissue (A and B) showed three distinctive layers, and in decellularized tissue, there was no evidence of the nucleus (C and D). Representative micrographs of native (E and F) and decellularized tissues (G and H) stained by Masson’s trichrome. Collagen fibers are demonstrated in blue color. The DNA content of control and decellularized samples was compared (I), and it significantly decreased in the decellularized group (^*^p < 0.05). The in vitro biodegradation test (J) indicated the significant differences between both studied groups (^*^p < 0.05). Based on the MTT assay, there was no significant difference in the optical density of both studied groups at 24 and 72 hours (K). The black and blue arrows show the epidermis and stroma of hypodermis, respectively

Microscopic observations of DST compared to the native group are shown in Figure 2. In H&E-stained tissue sections, the morphology of the epidermis, dermis, and hypodermis of native control skin were normal (Fig. 2A and 2B), whereas no nuclei was seen in the DST sections, and the epithelial cells were completely removed (Fig. 2C and 2D). However, the structure of the stroma (blue arrow) was well preserved in the DST. Masson’s trichrome staining showed that the collagen fibers (Fig. 2E and 2H) had similar arrangement and density in both decellularized (Fig. GE and 2H) and native control groups (Fig. 2E and 2F). 

**DNA content evaluations**

The amount of DNA in the control and the decellularized groups were 24.5 ± 3.7 ng/100 mg and 0.22 ± 0.09 ng/100 mg of wet tissue, respectively (Fig. 2I). In the decellularized samples, the amount of DNA content significantly decreased by 99.07% compared to the control group (*p* < 0.05). 

**Results of biodegradability evaluations**

The in vitro degradation of the decellularized scaffold compared to the control is summarized in Figure 2J. The percentages of biodegradability of the decellularized samples in the presence of enzyme at 24 and 48 h were 74.54 ± 0.001% and 92.56 ± 0.002%, and those in the control group treated with PBS were 20.08 ± 0.001% and 28.97± 0.002%, respectively. There was a significant difference between these groups regarding the percentage of biodegradation (*p* < 0.05).

**Outcomes of cell viability and cytotoxicity assessment**

The results of the MTT test after 24 and 72 h are summarized in Figure 2K. The optical densities in the control group after 24 and 72 h were 0.61 ± 0.04 and 0.67 ± 0.03, whereas this amount in the decellularized sample were 0.59 ± 0.02 and 0.76 ± 0.02 respectively, showing no significant difference between the two studied groups (*p* < 0.05).

**Raman confocal microscopy evaluations**

**Fig. 3:**
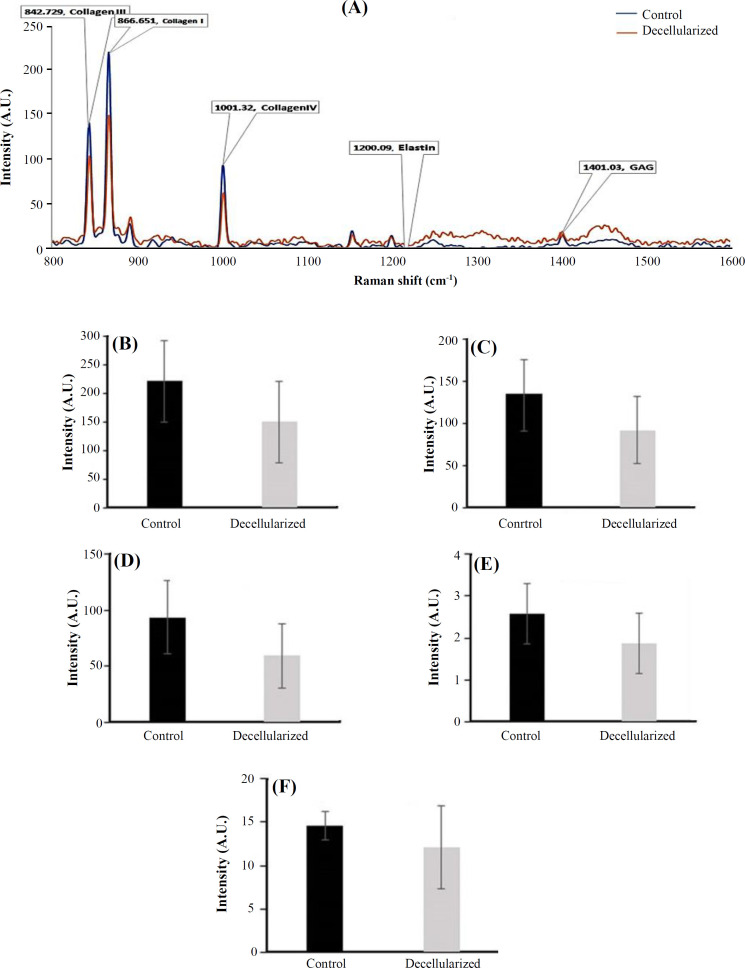
Raman confocal microscopy of the skin tissue for evaluating the amounts of extracellular components. Comparison of band intensity in the control and decellularized scaffold is demonstrated as 800-1600 cm^-1^ (A). The intensity of (B) type I collagen, (C) type III collagen, (D) type IV olagen, (E), elastin, and (F) glycosaminoglycan were compared between the control and decellularized groups. There was no significant difference between two groups regarding the intensity of the evaluated proteins

Representative diagrams of the peak intensity obtained from Raman confocal microscopy in native control and decellularized tissues are presented in Figure 3A. The peaks related to glycosaminoglycan, collagen I, collagen III, collagen IV, and elastin were considered to be in the range of 800-1600 cm^-1 ^spectra. There was a strong peak at the wavelength of 867, which is related to the dominant biochemical composition of collagen I, such as amino acid proline and hydroxyproline. A strong peak at 842 cm^-1 ^was found in both normal and decellularized tissues, relating to collagen III. In addition, a strong peak at 1001 cm^-1 ^is attributed to the amino acid phenylalanine, as a compound of collagen IV. The ranges of 1375-1410 cm^-1 ^and 1250-1340 cm^-1 ^were considered for glycosaminoglycans and elastin protein, respectively. Quantitative numbers of the mentioned ECM components in both studied groups were compared and summarized in Figure 3B-3F. No significant difference was observed between the control and decellularized groups (*p* > 0.05).

**Observation of the recellularized scaffold by light microscopic**

**Fig. 4:**
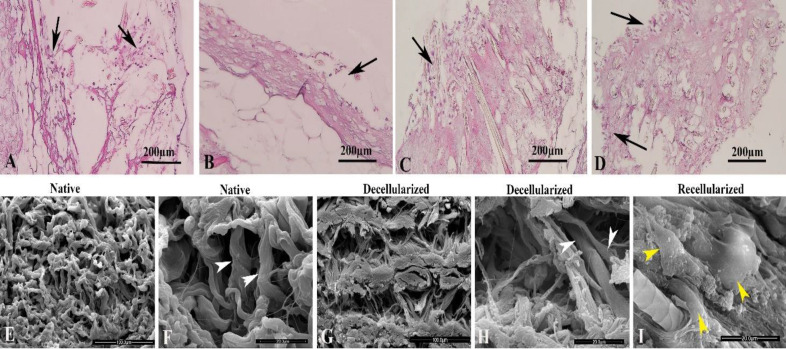
Photomicrographs of the recellularized skin scaffold stained with H&E. The micrographs show the cells (black arrows) are attached to different parts of the scaffold (A-D). The SEM micrographs of native (E and F) decellularized (G and H) and recellularized (I) scaffolds are demonstrated. The white arrowheads show a similar pattern of collagen in the studied groups (F and H). The yellow arrowheads show the fibroblast cells in recellularized tissue (I).

One week after culturing of MEFs on the decellularized scaffold, the micrographs obtained from the recellularized scaffolds (Fig. 4A-4D) showed that the MEFs attached and penetrated into different parts of the decellularized tissue. 

**SEM observation**

Representative SEM micrographs of de- and recellularized tissue are presented in Figure 4E-4I. The collagen fibers were observed in a discontinuous and irregular form in the native and decellularized tissue. Besides, a similar porous structure and fibers were found in the decellularized tissue before cell seeding (Fig. 4E-4H). The mean pore size in the native and decellularized scaffolds were 27 ± 0.10 and -28 ± 0.012 µm, respectively, which its difference was not significant. However, the image of the recellularized tissue showed several attached MEFs (Fig. 4I). 

**Gross observations of wound healing**

**Fig. 5:**
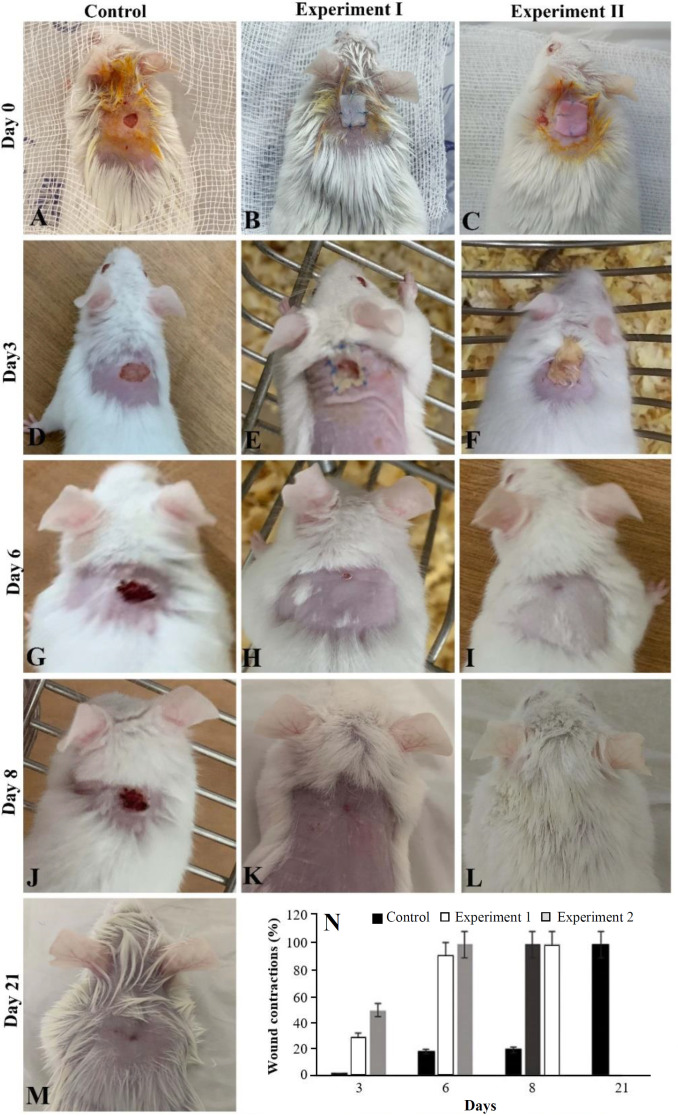
Comparison of wound healing observation in three studied groups (A-M) during 21 days. For photography, the cotton bandage used for covering wounds in all study groups was removed. The morphology of wound healing in the control, experimental group I, and experimental group II are shown in the first, second, and third columns, respectively. The wounds were covered by a recellularized scaffold in the experiment group I and by a decellularized scaffold in the experiment group II. The percentage of wound contraction was compared in all groups of study based on the area of the wound after the operation (N).

The gross observation of wound healing on the back of the male mice in three studied groups was presented in Figure 5A-5M. Based on these figures, all (100%) the mice showed the wound healed on day 21 in the control group. However, in the experimental group I, 33.3% of mice on day six and 66.6% on day eight were repaired. The percentage of wound healing on day eight was 100% in the experimental group II.

**Wound contraction**

The data of wound contraction are summarized in Figure 5N. The percentage of wound contraction on the third day was 2 ± 0.2% in the control group, 30 ± 0.5% in the experimental group I, and 50% ± 0.4 in the experimental group II. On day eight, these percentages were 20% ± 0.3%, 100%, and 100%, respectively. In the control group, the percentage of contraction on day 21 was 100%.

**Findings of histological evaluation and immunostaining of wound healing**

**Fig. 6:**
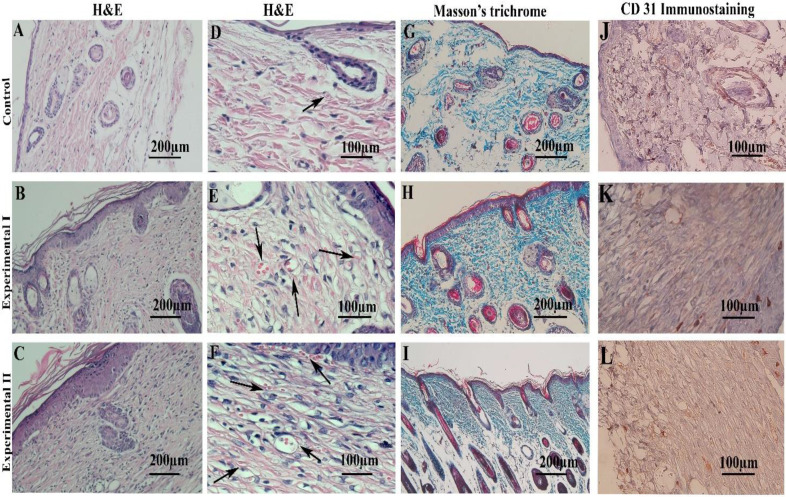
The light microscopic observation of recovered mouse skin tissue on the day of wound closing. The micrographs of skin tissue stained with H&E at low (first column, A-C) and high magnifications (second column, D-F) are demonstrated in the control group (A and D), experimental group I (B and E), and experimental group II (C-F). Black arrows show the small vessels. The tissue sections stained by Masson’s trichrome are indicated in the third column (G-I). The immunostaining of the CD 31 marker as an endothelial protein in studied groups is presented in the fourth column (J-L). The brown color demonstrates the positive reaction for this marker

H&E and Masson’s trichrome staining of control, experimental I, and experimental II after wound healing are shown in Figure 6. As shown in this Figure, the re- epithelialization and formation of three layers of skin was observed in the recovered tissue in both experimental groups, whereas the epidermis was thicker in both experimental groups than the control group (Fig. 6A-AC). Moreover, the presence of several small capillaries containing blood cells was prominent in both experimental groups (Fig. 6D-6F). Using Masson’s trichrome staining method, an increase in the density of collagen fiber was observed in both experimental groups compared to the control group (Fig. 6G-6I). Immunostaining of the recovered tissue showed a positive reaction for CD 31 antigen as an endothelial marker in the blood vessels of the studied tissue sections (Fig. 6J-6L).

**In vivo observation of** **Hoechst** **-labeled MEFs**

**Fig. 7:**
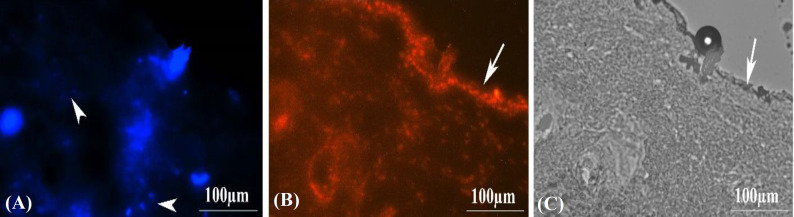
The morphology of Hoechst-labeled MEF cells in the recovered skin tissue in experimental group II has been observed under a fluorescent microscope. The labeled cells are colored blue (A; white arrowhead), and they are scattered within the tissue. (B) The cell nucleus was labelled using PI and demonstrated as orang color. (C) The phase contrast of the repair skin. White arrows show the epidermis

The presence of Hoechst-labeled cells in the recovered tissue was confirmed using a fluorescence microscope (Fig. 7). As illustrated in this figure, the labeled MEFS cells were observed as blue (donor cells), whereas all other cell nuclei stained with PI were observed as orange color.

## DISCUSSION

Studies have recently focused on decellularized tissue, because of its structural similarity to the normal extracellular matrix, to improve the design of wound dressings. This study is the first to investigate the process of wound healing by covering the decellularized skin scaffold with MEFs. 

In the first phase of the study, our morphological results using H&E and Masson’s trichrome staining showed that the applied decellularization method is efficient in removing cellular component and preserving the tissue structure and stroma similar to normal mouse skin. These results are related to the use of suitable detergents, including EDTA, SDS, and Triton X-100. The combination of these detergents is commonly used to create a cell-free scaffold ^[^^2^^-^^12^^]^. SDS as an ionic detergent, which is effective for cell removal with oreservation of the ECM intact, whereas Triton X-100 cannot remove the protein-protein bonds^[^^26^^-^^28^^]^. The concentration and duration of SDS treatment are two important factors in the process of de-cellularization^[^^26^^,^^27^^]^. In our applied protocol, enzymatic digestion has not been used, which may be an advantage for this method for influencing the mechanical or biochemical properties of the decellularized scaffold. The analysis of the DNA content showed that decellularization removes the maximum amount of genetic material, in which less than one percentage (0.93%) of DNA remains in the decellularized scaffolds compared to the control group, which are in agreement with other studies^[^^18^^,^^26^^,^^27^^]^. Moreover, our results obtained from the MTT test confirmed that the created DST was non-toxic, and it could improve cell proliferation after 72 h of culture. In agreement with these results, it was found that the efficiency of the decellularized scaffold obtained from different sources can be associated with good preservation of ECM and its non-toxic properties^[^^26^^,^^27^^]^. 

In the present study, for the first time, we co-cultured MEFs on the skin scaffold, and the results obtained by SEM and H&E staining showed that these cells were adhered and penetrated into the DST. The ECM component is critically involved in cell attachment via their receptors such as integrin^[^^29^^]^. In the line with this observation, Raman confocal microscopy revealed a favorable preservation of important binding molecules such as collagen types I, II, and IV and GAG in DST. In addition, the similar porosity of DST to the control could be suitable for growth, proliferation, and penetration of cells into the scaffold^[^^30^^]^. 

Our in vivo study demonstrated that the wound closure time in the experimental group I, which applied SDT with MEFs, was faster (day 6) than that of the group used only the decellularized scaffold (experiment group II). Moreover, in the control group, the duration of wound healing was extended to 21 days after the operation, which was longer than that of both experimental groups. These results confirmed the beneficial effect of the skin scaffold in the presence of MEFs for tissue repair. The combination of these two parameters in the experimental group I had positive effects on the rate and duration of wound healing. The healing process can be evaluated from different viewpoints, including the time of wound closure, tissue regeneration time, re-epithelization, collagen density, and formation of new capillaries. Our micrographs captured by fluorescence microscopy revealed some labeled MEFs in the recovered tissue of the experimental group I. This observation suggests that these cells are directly involved in skin repair by proliferation, as well as differentiation into keratinocytes or dermal cells. Fibroblasts, as multipotent and prominent cells of connective tissue, are contributed to the self-renewal, regeneration, and repair of tissue^[^^20^^,^^31^^-^^34^^]^. Fibroblasts with different sources, diverse appearances, and distinct activities synthesize and regenerate the ECM and accelerate wound healing^[^^31^^-^^34^^]^. The embryonic type is an immature form of fibroblasts that has shown some unique characteristics such as differentiation into several cell types^[^^18^^,^^19^^]^. In addition, MEFs migrate, proliferate and synthesize collagen fibers faster than their adult counterparts. Embryonic fibroblasts also reduce inflammatory responses during wound healing and decrease the time of wound healing^[^^31^^]^. Our results confirmed this statement by showing a faster repair time for the group that applied MEFs than that of other studied groups. Moreover, the morphological and immune-histochemical staining displayed that experimental group I had prominent vascular beds in the recovered tissue, which proposes that MEFs can accelerate the neovascularization and formation of new capillaries to provide nutrients and growth factors for tissue regeneration. MEFs may accelerate angiogenesis by differentiation into endothelial lineages or secreting angiogenic factors. The in vivo experiments of Lin et al. also revealed that the application of adipose-derived stem cells and dermal ECM hydrogel significantly accelerate repairing the rat skin by inducing the regeneration of vascularized skin^[^^17^^]^. Therefore, the recellularization of skin scaffolds using MEFs could have applications in tissue engineering and regenerative medicine.

## CONCLUSION

Using MEFs with decellularized skin as a wound dressing increases the rate of wound healing and also formation of new capillaries. This system may be beneficial for clinical applications in the field of tissue engineering.

## DECLARATIONS

### Acknowledgments

 This research project was extracted from M.Sc. thesis, which was supported by Tarbiat Modares University, Tehran, Iran. In this manuscript, AI has not been recruited in all processes of this work.

### Ethical approval

All the experimental procedures in this study were conducted in accordance with the Institutional Animal Care guidelines of Trabiat Modares University and approved by the Research Ethics Committee of Tarbiat Modares University, Tehran, Iran (ethical code: R.MODARES.AEC.1401.028).

### Consent to participate

Not applicable.

### Consent for publication

All authors reviewed the results and approved the final version of the manuscript.

### Authors’ contributions

AG: performed the experiments; HB: co-supervised and involved in protocol development; SP: involved in molecular experiments; MS: supervised the study and performed the writing of the manuscript.

### Data availability

 All relevant data can be found within the manuscript. 

### Competing interests

The authors declare that they have no competing interests. 

### Funding

This research received no specific grant from any funding agency in the public, commercial, or not-for-profit sectors.

### Supplementary information

The online version does not contain supplementary material.
